# Progress Toward Hepatitis B Control — World Health Organization European Region, 2016–2019

**DOI:** 10.15585/mmwr.mm7030a1

**Published:** 2021-07-30

**Authors:** Nino Khetsuriani, Liudmila Mosina, Pierre Van Damme, Antons Mozalevskis, Siddhartha Datta, Rania A. Tohme

**Affiliations:** ^1^Global Immunization Division, Center for Global Health, CDC; ^2^Vaccine-Preventable Diseases and Immunization Programme, World Health Organization Regional Office for Europe, Copenhagen, Denmark; ^3^Faculty of Medicine and Health Sciences, University of Antwerp, Antwerp, Belgium; ^4^HIV and Viral Hepatitis Unit, World Health Organization Regional Office for Europe, Copenhagen, Denmark.

In 2019, an estimated 14 million persons in the World Health Organization (WHO) European Region* (EUR) were chronically infected with hepatitis B virus (HBV), and approximately 43,000 of these persons died from complications of chronic HBV infection (*1*). In 2016, the WHO Regional Office for Europe set hepatitis B control program targets for 2020, including 1) ≥90% coverage with 3 doses of hepatitis B vaccine (HepB3), 2) ≥90% coverage with interventions to prevent mother-to-child transmission (MTCT) of HBV,^†^ and 3) ≤0.5% prevalence of HBV surface antigen (HBsAg)^§^ in age groups eligible for vaccination with hepatitis B vaccine (HepB) (*2*–*4*). This report describes the progress made toward hepatitis B control in EUR during 2016–2019. By December 2019, 50 (94%) of 53 countries in EUR provided routine vaccination with HepB to all infants or children aged 1–12 years (universal HepB), including 23 (43%) countries that offered hepatitis B birth dose (HepB-BD) to all newborns. In addition, 35 (73%) of the 48 countries with universal infant HepB vaccination reached ≥90% HepB3 coverage annually during 2017–2019, and 19 (83%) of the 23 countries with universal birth dose administration achieved ≥90% timely HepB-BD coverage^¶^ annually during that period. Antenatal hepatitis B screening coverage was ≥90% in 17 (57%) of 30 countries that selectively provided HepB-BD to infants born to mothers with positive HBsAg test results. In January 2020, Italy and the Netherlands became the first counties in EUR to be validated to have achieved the regional hepatitis B control targets. Countries can accelerate progress toward hepatitis B control by improving coverage with HepB and interventions to prevent MTCT and documenting achievement of the HBsAg seroprevalence target through representative serosurveys or, in low-endemicity countries, antenatal screening.

## Immunization Activities

As a major intervention to prevent perinatal and childhood hepatitis B infections, WHO recommends that all infants receive ≥3 doses of HepB, including a timely birth dose (*5*). Most countries in EUR introduced HepB vaccination >15 years ago (Table 1). Countries report information on immunization schedules and coverage annually to WHO and UNICEF using the WHO/UNICEF Joint Reporting Form. WHO and UNICEF review administrative coverage data and surveys to generate country-specific coverage estimates.**

**TABLE 1:** Year of introduction of hepatitis B vaccine, hepatitis B vaccine routine vaccination and birth dose policies, vaccination schedule, coverage with a third dose of hepatitis B vaccine and a timely hepatitis B birth dose, and antenatal screening for hepatitis B virus infection — World Health Organization European Region, 2016–2019

Country (year of HepB introduction*^,†^)	HepB vaccination policy*^,§^		2019 HepB schedule*	HepB3 coverage,* %					Timely HepB-BD coverage,* %					Antenatal screening	
	Infant/Childhood	At birth		Year				≥90% each year, 2017–2019^¶^	Year				≥90% each year, 2017–2019^¶^	In place**	Coverage, %^††^
				2016	2017	2018	2019		2016	2017	2018	2019			
Albania (1994)	Universal	Universal	B, 2, 4, 6 mos	98	99	99	99	Yes	99	99	99	99	Yes	—	—
Andorra (1997)	Universal	Selective	2, 4, 12 mos	94	98	98	98	Yes	NA	NA	NA	NA	NA	Yes	NR
Armenia (2000)	Universal	Universal	B, 6, 12, 18 wks, 18 mos	94	94	92	92	Yes	98	97	97	96	Yes	—	—
Austria (1997)	Universal	Selective	2, 4, 10 mos	87	90	85	85	No	NA	NA	NA	NA	NA	Yes	NR
Azerbaijan (2001)	Universal	Universal	B, 2, 3, 4 mos	97	95	95	94	Yes	99	99	99	98	Yes	—	—
Belarus (1996)	Universal	Universal	B, 2, 3, 4 mos	96	98	98	97	Yes	98	98	98	98	Yes	—	—
Belgium (1996)	Universal	Selective	8, 12, 16 wks, 15 mos	97	97	97	97	Yes	NA	NA	NA	NA	NA	Yes	80–85
Bosnia and Herzegovina (2001)	Universal	Universal	B, 1, 6 mos	78	77	80	80	No	NR	NR	NR	NR	NR	—	—
Bulgaria (1991)	Universal	Universal	B, 1, 6 mos; B, 2, 3, 4 mos	91	92	85	85	No	96	97	96	96	Yes	—	—
Croatia (1999)	Universal	Selective	2, 4, 6, 18 mos	92	92	93	93	Yes	NA	NA	NA	NA	NA	Yes	>90
Cyprus (1989)	Universal	Selective	2, 4, 8–12 mos	97	97	97	94	Yes	NA	NA	NA	NA	NA	Yes	NR
Czechia (2001)	Universal	Selective	3, 5, 11 mos	96	94	96	97	Yes	NA	NA	NA	NA	NA	Yes	100
Denmark (2009)**^§§^**	Selective	Selective	—	NA	NA	NA	NA	NA	NA	NA	NA	NA	NA	Yes	99.9
Estonia (2003)	Universal	Selective	3, 4.5, 6 mos, 2 yrs	93	92	93	91	Yes	NA	NA	NA	NA	NA	Yes	>90
Finland (1993)^§§^	Selective	Selective	—	NA	NA	NA	NA	NA	NA	NA	NA	NA	NA	Yes	97.8
France (1994)	Universal	Selective	2, 4, 11 mos	90	90	91	91	Yes	NA	NA	NA	NA	NA	Yes	92.4
Georgia (2001)	Universal	Universal	B, 2, 3, 4 mos	92	91	93	94	Yes	94	94	97	94	Yes	—	—
Germany (1995)	Universal	Selective	2, 3, 4, 11–14 mos	87	87	87	87	No	NA	NA	NA	NA	NA	Yes	>90
Greece (2000)	Universal	Selective	2, 4, 6–18 mos	96	96	96	96	Yes	NA	NA	NA	NA	NA	Yes	91.3
Hungary (1999)^¶¶^	Universal	Selective	12 yrs	NR	NR	NR	NR	NR	NA	NA	NA	NA	NA	Yes	90
Iceland (2011)^§§^	Selective	Selective	—	NA	NA	NA	NA	NA	NA	NA	NA	NA	NA	Yes	50
Ireland (2008)	Universal	Selective	2, 4, 6 mos	95	95	94	93	Yes	NA	NA	NA	NA	NA	Yes	>95
Israel (1998)	Universal	Universal	B, 1, 6 mos	95	97	96	96	Yes	95	95	95	95	Yes	—	—
Italy (1982)	Universal	Selective	2, 4, 11 mos	93	94	95	95	Yes	NA	NA	NA	NA	NA	Yes	97.7
Kazakhstan (1998)	Universal	Universal	B, 2, 4 mos	82	99	98	97	Yes	95	90	95	93	Yes	—	—
Kyrgyzstan (2001)	Universal	Universal	B, 2, 3.5, 5 mos	96	92	94	95	Yes	96	97	97	96	Yes	—	—
Latvia (1997)	Universal	Selective	2, 4, 6, 12–15 mos	98	98	96	99	Yes	NA	NA	NA	NA	NA	Yes	88
Lithuania (1998)	Universal	Universal	B, 1, 6 mos	95	94	93	92	Yes	97	97	97	97	Yes	—	—
Luxembourg (2003)	Universal	Selective	2, 3, 13 mos	94	94	96	96	Yes	NA	NA	NA	NA	NA	Yes	95
Malta (2005)	Universal	Selective	12, 13, 18 mos	97	88	98	98	No	NA	NA	NA	NA	NA	Yes	100
Moldova (1995)	Universal	Universal	B, 2, 4, 6 mos	90	89	94	94	No	99	96	96	93	Yes	—	—
Monaco (1994)	Universal	Selective	2, 4, 11 mos	99	99	99	99	Yes	NA	NA	NA	NA	NA	Yes	NR
Montenegro (2006)	Universal	Selective	9, 13 wks, 9 mos	75	73	72	72	No	NA	NA	NA	NA	NA	No	—
Netherlands (2011)***	Universal	Selective	2, 3, 4–11 mos	93	92	92	92	Yes	NA	NA	NA	NA	NA	Yes	99
North Macedonia (2004)	Universal	Universal	B, 2, 6 mos	94	91	92	92	Yes	98	98	98	98	Yes	—	—
Norway (2017)***	Universal	Selective	3, 5, 12 mos	NA	NA	NR	96	ID	NA	NA	NA	NA	NA	Yes	NR
Poland (1997)	Universal	Universal	B, 7–8 wks, 7 mos	95	93	91	91	Yes	93	93	93	93	Yes	—	—
Portugal (1994)	Universal	Universal	B, 2, 6 mos	98	98	98	98	Yes	97	97	97	97	Yes	—	—
Romania (1995)	Universal	Universal	B, 2, 4, 11 mos	90	92	93	90	Yes	93	36	68	99	No	—	—
Russia (2000)	Universal	Universal	B, 1, 6 mos	97	97	97	97	Yes	NR	NR	NR	NR	NR	—	—
San Marino (1995)	Universal	Selective	3, 5, 11 mos	86	82	78	87	No	NA	NA	NA	NA	NA	Yes	100
Serbia (2006)	Universal	Universal	B, 4 wks, 10 mos	91	93	91	94	Yes	99	99	99	99	Yes	—	—
Slovakia (1997)	Universal	Selective	2, 4, 10 mos	96	96	96	97	Yes	NA	NA	NA	NA	NA	Yes	NR
Slovenia (1998)^¶¶,†††^	Universal	Selective	5 yrs (2 doses), 6 yrs	88	89	87	88	No	NA	NA	NA	NA	NA	Yes	NR
Spain (1996)	Universal	Selective	2, 4, 11 mos	97	95	96	96	Yes	NA	NA	NA	NA	NA	Yes	NR
Sweden (2016)	Universal	Selective	3, 5, 12 mos	67	76	92	97	No	NA	NA	NA	NA	NA	Yes	NR
Switzerland (2018)^¶¶,§§§^	Universal	Selective	2, 4, 12 mos; 11–15 yrs, +6 mos	69	69	96	96	ID	NA	NA	NA	NA	NA	Yes	97
Tajikistan (2002)	Universal	Universal	B, 2, 3, 4 mos	97	96	96	97	Yes	92	99	99	99	Yes	—	—
Turkey (1998)	Universal	Universal	B, 1, 6 mos	98	96	98	99	Yes	99	99	99	99	Yes	—	—
Turkmenistan (2002)	Universal	Universal	B, 2, 3, 4 mos	98	99	99	99	Yes	99	99	99	99	Yes	—	—
Ukraine (2003)	Universal	Universal	8, 12, 16 wks	26	52	67	76	No	37	49	60	60	No	—	—
UK (2017)***	Universal	Selective	B, 2, 6 mos	NA	NA	NR	93	ID	NA	NA	NA	NA	NA	Yes	>95
Uzbekistan (2001)	Universal	Universal	B, 2, 3, 4 mos	99	99	98	96	Yes	99	99	95	99	Yes	—	—
**European Region^¶¶¶^**	**—**	**—**	**—**	**82**	**84**	**84**	**92**	**—**	**66**	**65**	**67**	**68**	**—**	**—**	**—**

**Abbreviations:** B = birth; DTaP-Hib-HepB-IPV = hexavalent vaccine containing diphtheria and tetanus toxoids, acellular pertussis, *Haemophilus influenzae* type b, hepatitis B, and inactivated poliovirus components; HBsAg = hepatitis B virus surface antigen; HepB = hepatitis B vaccine; HepB3 = third dose of HepB; HepB-BD = birth dose of HepB; ID = insufficient data to determine (no reports for 1 or 2 years); NA = not applicable; NR = not reported; UK = United Kingdom; +6 mos = 6 months after the previous dose.

* https://immunizationdata.who.int

^†^ Introduction of universal infant HepB vaccination into national immunization schedules. Exceptions: HepB was introduced regionally or subnationally before nationwide introduction in the following countries: Bosnia and Herzegovina (1999), Estonia (1999), Georgia (2000), Kyrgyzstan (1999), Poland (1995), Serbia (2002), Spain (1991), Sweden (2014), Ukraine (2001), and Uzbekistan (1997).

^§^ HepB vaccination policy: universal = all persons in the applicable age group (i.e., all infants, children aged 1–12 years, or adolescents aged 13–15 years for routine HepB vaccination, and all newborns for HepB-BD) receive HepB; selective = only infants born to mothers with positive HBsAg test results receive HepB vaccination, starting with HepB-BD.

^¶^ Two of the criteria for validation of hepatitis B control are 1) to achieve HepB3 coverage ≥90% for the 3 preceding years, applicable only to countries with universal infant HepB vaccination policy and 2) to achieve timely HepB-BD coverage ≥90% for the 3 preceding years, applicable only to countries with universal HepB-BD policy.

** A criterion for validation of hepatitis B control, applicable only to countries with selective HepB-BD policy; data for other countries not included. Sources: WHO 2018 Regional Office for Europe survey (Andorra, Austria, Belgium, Czechia, Estonia, France, Germany, Hungary, Ireland, Italy, Latvia, Malta, Monaco, Montenegro, the Netherlands, Norway, San Marino, Slovakia, Spain, and Switzerland); https://apps.who.int/iris/bitstream/handle/10665/85397/9789241564632_eng.pdf?sequence=1 (Cyprus, Denmark, Finland, Iceland, Luxembourg, Slovenia, and Sweden); reports submitted to the Regional Hepatitis B Working Group (Croatia and UK); https://europepmc.org/article/PMC/1475591 (Greece).

^††^ A criterion for validation applicable only to countries with selective HepB-BD policy. Sources: 2018 WHO Regional Office for Europe survey (Czechia, Germany, Italy, Latvia, San Marino, and Switzerland); https://www.ecdc.europa.eu/sites/default/files/media/en/publications/Publications/antenatal-screening-HIV-hepatitis-B-syphilis-rubella-EU.pdf (Belgium, Estonia, Hungary, Iceland, Ireland, Luxembourg, and Malta); reports submitted to the Regional Hepatitis B Working Group (Croatia, the Netherlands, and UK); https://en.ssi.dk/surveillance-and-preparedness/surveillance-in-denmark/annual-reports-on-disease-incidence/pregnancy-screening-2019 (Denmark); https://www.julkari.fi/bitstream/handle/10024/114883/URN_ISBN_978-952-302-057-3.pdf?sequence = 1&isAllowed = y (Finland); http://beh.santepubliquefrance.fr/beh/2015/15-16/pdf/2015_15-16_4.pdf (France); https://europepmc.org/article/PMC/1475591 (Greece).

**^§§^** Denmark, Finland, and Iceland do not have universal HepB in their routine childhood immunization schedules but selectively vaccinate only infants born to mothers with positive HBsAg test results.

^¶¶^ Vaccination of older children or adolescents (Hungary, 12 years; Slovenia, 5–6 years; Switzerland, 11–15 years during 1997–2018, before switching to universal infant HepB immunization).

*** HepB was given only to infants of mothers with positive HBsAg test results before transition to universal infant vaccination in the Netherlands (2002–2010), Norway (2002–2016), and UK (2001– mid-2017).

^†††^ Slovenia does not vaccinate infants against HepB; therefore, WHO/UNICEF estimates are not generated. Instead, country-reported official HepB3 coverage among children aged 6 years is included.

^§§§^ In Switzerland, reported coverage with HepB for adolescents until 2018. Since 2018, WHO/UNICEF estimates of coverage with the third dose of hepatitis B-containing hexavalent vaccine (DTaP-Hib-HepB-IPV) among infants (reported as DTP3).

^¶¶¶^ A weighted sum of WHO/UNICEF estimates of national coverage by target population from the United Nations Population Division's World Population Prospects for all 53 countries of the region. HepB3 coverage includes all 53 countries in EUR. HepB-BD coverage includes 23 countries that implement universal birth dose policy. Two countries, Bosnia and Herzegovina and Russia, do not report HepB-BD coverage, but their population is included in the denominator, resulting in lower coverage in this group than in most individual countries with reported coverage data.

In 2019, 48 (91%) of the 53 countries in EUR provided universal routine infant HepB vaccination, two^††^ (4%) (Hungary and Slovenia) provided universal routine HepB vaccination to children aged 5–12 years, and three countries (6%) (Denmark, Finland, and Iceland) implemented selective HepB vaccination, only immunizing those born to mothers with positive HBsAg test results.^§§^ Twenty-three (43%) countries provided HepB-BD to all newborns, and 30 (57%) provided HepB-BD selectively to children born to mothers with positive HBsAg test results. During 2016–2019, regional HepB3 coverage increased from 82% to 92%, partly because three more countries (Norway, Switzerland, and the United Kingdom)^¶¶^ introduced universal infant HepB vaccination during 2017–2018. Among the countries that provided universal infant HepB vaccination, those that reported ≥90% HepB3 coverage among infants increased from 37 (82%) of 45 countries during 2016–2017 to 41 (85%) of 48 countries in 2019. However, HepB3 coverage was <90% for ≥3 years during 2016–2019 in six countries.*** Of the 21 countries with universal HepB-BD that reported birth dose coverage to WHO,^†††^ coverage with timely HepB-BD during 2016–2019 was ≥90% in 2019–2020 (90%–95%). 

## Antenatal Screening and Postexposure Prophylaxis

The 30 countries that implement a selective HepB-BD policy aim to prevent MTCT of HBV infection through antenatal HBV screening combined with postexposure prophylaxis of infants born to mothers with positive HBsAg test results. Information on implementation of these interventions is not routinely reported to WHO. Based on the responses to a survey conducted by the WHO Regional Office for Europe in 2018^§§§^ and published reports, 29 (97%) of those 30 countries^¶¶¶^ conducted nationwide antenatal screening for HBsAg (Table 1). Antenatal screening coverage data were available for 20 (69%) of these countries, and 17 (85%) reported ≥90% coverage.**** Among infants born to HBV-infected mothers in these countries, immunization coverage data were available for HepB-BD in nine (31%) countries^††††^ and for HepB3 in five (17%)^§§§§^ countries. HepB-BD coverage exceeded 90% in all nine countries, and HepB3 coverage exceeded 90% in four of five countries.

## HBsAg Seroprevalence

Because most chronic HBV infections are asymptomatic, particularly among young children, the impact of hepatitis B vaccination is assessed based on the HBsAg seroprevalence among children (*6*). However, in EUR, because of early regional introduction of HepB, the age group for serosurveys for validation purposes is defined as cohorts eligible for HepB vaccination. For EUR countries with low endemicity before vaccine introduction (prevaccine), where conducting large-scale hepatitis B serosurveys might not be justified, HBsAg seroprevalence of ≤0.5% among pregnant women is considered acceptable evidence that the seroprevalence target was achieved.

By December 2019, representative nationwide or regional serosurveys have demonstrated ≤0.5% HBsAg seroprevalence in at least one vaccinated or partially vaccinated age group in five countries and in a prevaccine cohort in one country (the Netherlands) (Table 2). Serosurveys initiated recently for validation purposes in several countries, in some cases with support from WHO and other international partners, have been put on hold because of the COVID-19 pandemic. HBsAg seroprevalence of ≤0.5% among pregnant women has been reported from nine (36%) of 25 countries with low endemicity, sometimes with higher prevalence among foreign-born women than among women who were not foreign-born (e.g., Denmark, Italy, and the Netherlands) (Table 2).

**TABLE 2:** Hepatitis B virus surface antigen seroprevalence based on representative population-based serosurveys or among pregnant women during antenatal screening in selected countries — World Health Organization European Region, 2003–2019

Country	Year	Geographic area	Age group, yrs (sample size)	Vaccination status*	HBsAg prevalence, % (95% CI)^†^
**Population-based representative serosurveys**					
Germany^§^	2008–2011	Nationwide	≥18 (7,047)	Prevaccine and partially vaccinated	0.3 (0.2–0.6)
Netherlands^¶^	2007	Nationwide	0–79 (6,246)	Prevaccine	Overall, 0.2 (0.1–0.4)
					Dutch, 0.1 (0.0–0.4)
					Foreign-born, 1.1 (0.4–2.7)
Portugal**	2012–2014	Nationwide	≥18 (1,685)	Pre- and postvaccine	Post-vaccine (18–34 yrs), 0.1 (NR)
Spain^††^	2017–2018	Nationwide	2–80 (6,056)	Pre- and postvaccine	Post-vaccine (2–19 yrs), 0 (NR)
					Combined pre-and postvaccine (20–80 yrs), 0.22 (0.10–0.35)
Tajikistan^§§^	2010	Nationwide	1–24 (2,188)	Pre- and postvaccine	Postvaccine (1–6 yrs), 0.4 (0.1–1.3)
**Among pregnant women (in countries with selective hepatitis B birth dose vaccination policy)**					
Croatia^¶¶^	2016–2018	Nationwide	NA	NA	<0.2
Denmark***	2019	Nationwide	NA	NA	Overall, 0.25
					Danish-born, <0.01
					Foreign-born, 0.25
Finland^†††^	2005–2009	Nationwide	NA	NA	0.13
Ireland^§§§^	2004–2009	Western Ireland	NA	NA	0.21
Italy^¶¶¶^	2008–2009	Twelve regions	NA	NA	Overall, 0.86
					Italian-born, 0.4
					Foreign-born, 2.5
Netherlands****	2012–2016	Nationwide	NA	NA	0.3
Norway^††††^	2003–2004	Northern Norway	NA	NA	0.1
Spain^§§§§^	2015	Nationwide	NA	NA	0.42
UK^¶¶¶¶^	2015	England	NA	NA	0.4

**Abbreviations:** CI = confidence interval; HBsAg = hepatitis B virus surface antigen; HepB = hepatitis B vaccine; NA = not applicable; NR = not reported; UK = United Kingdom.

* Postvaccine = age groups eligible for vaccination with HepB; prevaccine = age groups not eligible for HepB vaccination; partially vaccinated = age groups in which some people were vaccinated before nationwide introduction of routine childhood HepB vaccination; combined pre- and postvaccine = age group for which estimates are provided include both pre- and postvaccine cohorts.

**^†^** Applicable to population-based serosurveys only.

^§^ https://edoc.rki.de/bitstream/handle/176904/1530/221UAZ0QXaYVg.pdf?sequence=1&isAllowed = y

^¶^ https://www.cambridge.org/core/services/aop-cambridge-core/content/view/0A7381A4CB391EE13C5028444DCEDA91/S095026881100224Xa.pdf/prevalence_of_hepatitis_b_virus_infection_in_the_netherlands_in_1996_and_2007.pdf

** https://journals.lww.com/eurojgh/Fulltext/2016/06000/Hepatitis_B_and_C_prevalence_in_Portugal_.5.aspx

^††^ https://www.mscbs.gob.es/profesionales/saludPublica/prevPromocion/vacunaciones/comoTrabajamos/docs/EstudioSeroprevalencia_EnfermedadesInmunoprevenibles.pdf

^§§^ https://www.sciencedirect.com/science/article/pii/S0264410X15007665

^¶¶^ The report submitted to the WHO European Regional Hepatitis B Working Group.

*** https://en.ssi.dk/surveillance-and-preparedness/surveillance-in-denmark/annual-reports-on-disease-incidence/pregnancy-screening-2019

^†††^ https://www.julkari.fi/bitstream/handle/10024/114883/URN_ISBN_978-952-302-057-3.pdf?sequence = 1&isAllowed = y

^§§§^ http://archive.imj.ie//ViewArticleDetails.aspx?ContentID = 3961

**^¶¶¶^** https://doi.org/10.1016/j.jinf.2010.11.014

**** The report submitted to the WHO European Regional Hepatitis B Working Group.

^††††^ Kristiansen MG, Eriksen BO, Maltau JM, et al. Prevalences of viremic hepatitis C and viremic hepatitis B in pregnant women in Northern Norway. Hepato-Gastroenterology 2009;56:1141–5.

^§§§§^ https://journals.plos.org/plosone/article?id=10.1371/journal.pone.0233528

^¶¶¶¶^ https://assets.publishing.service.gov.uk/government/uploads/system/uploads/attachment_data/file/583576/hpr0217_naism.pdf

## Validation

The Hepatitis B Regional Working Group of the European Technical Advisory Group of Experts was established in 2017 and developed the framework and criteria for validation of achievement of the regional hepatitis B control targets for countries in EUR (Table 3). The validation process was initiated in 2019. In 2019, the regional validation criteria for immunization coverage were met for HepB3 by 35 (73%) of 48 countries providing universal infant HepB vaccination and for timely HepB-BD by 19 (83%) of 23 countries implementing universal newborn vaccination, including 17 (32%) countries that met both criteria (Table 1). In January 2020, Italy and the Netherlands were validated to have achieved the regional hepatitis B control targets. The United Kingdom received conditional validation based on fully meeting the MTCT prevention and seroprevalence criteria (in antenatal screening), and by partially meeting immunization coverage criteria pending availability of 3 full years of data.^¶¶¶¶^ Croatia received conditional validation pending clarification of methods for assessing coverage with MTCT prevention interventions.

**TABLE 3:** Criteria for country validation of the achievement of the regional hepatitis B control targets, according to the Hepatitis B Regional Working Group, European Technical Advisory Group of Experts — World Health Organization European Region

Area of assessment*	Criteria	Comment
Routine hepatitis B immunization coverage	≥90% coverage for infants with ≥3 doses of hepatitis B vaccine (according to national immunization schedule)	For countries that implement universal hepatitis B vaccination; in each of the last 3 years
Prevention of mother-to-child transmission of hepatitis B virus	≥90% coverage with timely hepatitis B birth dose vaccination	For countries that implement universal newborn vaccination; in each of the last 3 years
	≥90% coverage with hepatitis B screening in pregnant women and ≥90% coverage with postexposure prophylaxis in infants born to infected mothers^†^	For countries that implement selective hepatitis B birth dose policy; in each of the last 3 years, if the data are routinely collected; one time, if based on a special assessment
HBsAg seroprevalence	≤0.5% HBsAg prevalence in cohorts eligible for vaccination	Required for countries with high and intermediate pre-vaccine endemicity of hepatitis B^§^
	≤0.5% HBsAg prevalence among pregnant women	Alternative criterion acceptable only for countries with historically low endemicity of hepatitis B

**Abbreviation:** HBsAg = Hepatitis B surface antigen.

* For a country to receive validation, the applicable criteria should be met in all three areas.

^†^ Includes administration of hepatitis B vaccine within 24 hours of birth, followed by ≥2 additional doses (according to national schedule); coverage targets apply to birth dose and HepB3.

^§^ Hepatitis B endemicity levels based on the prevalence of HBsAg: low (<2.0%), intermediate (2.0%–7.9%), and high (>8.0%).

## Discussion

During 2016–2019, EUR made substantial progress toward achieving hepatitis B control, resulting in validation of the first two countries (Italy and the Netherlands) and conditional validation of two other countries (Croatia and the United Kingdom). This progress is supported by a recent modeling study, which demonstrated 0.1% HBsAg seroprevalence among children aged 5 years in EUR (*3*). Among the 49 countries that have not yet initiated the validation process, 17 (74%) of 23 with a universal HepB-BD policy have met the HepB3 coverage and HepB-BD coverage criteria, and six (23%) of 26 countries with a selective birth dose policy met HepB3 coverage and antenatal screening coverage targets. Eight (16%) of these 49 countries met the ≤0.5% HBsAg seroprevalence target.

To accelerate validating achievement of the regional hepatitis B control target in EUR, some countries could consider submitting available documentation for validation, whereas others still need to generate the evidence required for validation. Although conducting nationally representative hepatitis B serosurveys might be challenging, and because the COVID-19 pandemic has further challenged their implementation, hepatitis B testing can be incorporated into other nationally representative serosurveys, including COVID-19 serosurveys, where feasible.

The historic differences in HBsAg prevalence and the diversity of HepB immunization strategies across EUR necessitated a differential approach to validation of hepatitis B control depending on national prevaccine endemicity and HepB vaccination policies. Although HepB3 immunization coverage is high in most countries, it remains consistently <90% in six countries, reflecting challenges in their immunization services. Countries can address these challenges by 1) providing sufficient support to national immunization programs to strengthen immunization systems, 2) monitoring public perception toward vaccinations and developing tailored strategies to create demand for vaccination among all population groups, and 3) strengthening immunization information systems to improve quality and availability of coverage data (*6*–*8*). The two countries in EUR with universal birth dose policy that currently do not report HepB-BD coverage (Bosnia and Herzegovina and Russia) will need to establish systems for monitoring and reporting birth dose coverage.

In countries that provide selective HepB-BD vaccination, establishing systems for continual monitoring of coverage with antenatal screening and HBsAg-positivity among pregnant women and of coverage with HepB-BD and HepB3 among exposed infants is needed to provide reliable data on seroprevalence and interventions to prevent MTCT of HBV for validation purposes. Available seroprevalence data showed a much higher prevalence of hepatitis B among foreign-born populations in several countries in EUR. Ensuring access to MTCT prevention measures for underserved populations, including immigrants, ethnic minorities, and other vulnerable groups, can help mitigate the impact of increased migration from high- and intermediate-endemicity areas on HBsAg prevalence in low-endemicity countries (*9*).

The findings in this report are subject to at least three limitations. First, missing HepB-BD coverage data for Bosnia and Herzegovina and Russia prevent determining whether these countries have met the HepB-BD coverage target. Second, timely HepB-BD coverage estimates might not be accurate for countries that do not monitor timeliness of HepB-BD administration. Finally, some HBsAg seroprevalence estimates were obtained >15 years ago and might not reflect the current prevalence in cohorts eligible for vaccination.

Despite progress made during 2016–2019, achieving the 2020 hepatitis B control goal in EUR will require programmatic improvements in underperforming countries. To accelerate the validation process, most countries will need to generate additional evidence of having achieved the regional targets. Some low- and middle-income countries will require continued external support to conduct serosurveys. Further, the COVID-19 pandemic has caused disruptions in immunization services and led to delays in implementation of serosurveys. Implementing the regional guidance on interventions to mitigate the impact of COVID-19 on immunization programs can help countries maintain or improve HepB vaccination coverage and accelerate progress toward the regional goal (*10*).

**Summary**

**What is already known about this topic?**

In 2019, 14 million persons in the World Health Organization European Region (EUR) were chronically infected with hepatitis B virus.

**What is added by this report?**

During 2016–2019, EUR made substantial progress towards hepatitis B control. Of 53 countries in EUR, 35, 19, and 17 countries met coverage targets for 3 doses of hepatitis B vaccine, the birth dose, and for hepatitis B screening of pregnant women, respectively. Two countries (Italy and the Netherlands) have achieved hepatitis B control.

**What are the implications for public health practice?**

Improving hepatitis B vaccination coverage, screening of pregnant women, and conducting hepatitis B seroprevalence assessments can help EUR to accelerate progress and document achievement of hepatitis B control targets.

## Footnotes

* EUR is one of six WHO regions and consists of the following 53 member states (total population, approximately 932 million): Albania, Andorra, Armenia, Austria, Azerbaijan, Belarus, Belgium, Bosnia and Herzegovina, Bulgaria, Croatia, Cyprus, Czechia, Denmark, Estonia, Finland, France, Georgia, Germany, Greece, Hungary, Iceland, Ireland, Israel, Italy, Kazakhstan, Kyrgyzstan, Latvia, Lithuania, Luxembourg, Malta, Moldova, Monaco, Montenegro, Netherlands, North Macedonia, Norway, Poland, Portugal, Romania, Russia, San Marino, Serbia, Slovakia, Slovenia, Spain, Sweden, Switzerland, Tajikistan, Turkey, Turkmenistan, Ukraine, United Kingdom, and Uzbekistan.

† In EUR, interventions to prevent MTCT of HBV include either 1) administering a timely birth dose of HepB vaccine to all newborns (universal birth dose policy) or 2) conducting routine antenatal screening of pregnant women for hepatitis B and vaccinating infants born to HBV-infected mothers with HepB birth dose within 24 hours of birth (selective birth dose policy), either of which is followed by ≥2 additional vaccine doses according to the national immunization schedule. In addition, some countries provide antiviral treatment to pregnant women with positive HBsAg test results and administer hepatitis B immune globulin at birth to infants of infected mothers.

§ Before introduction of vaccination, the HBV endemicity in EUR, defined by HBsAg antigen seroprevalence, ranged widely from low (<2.0%) in 25 countries, to intermediate (2.0%–7.9%) in 25 countries, to high (≥8.0%) in three countries.

¶ A timely HepB birth dose is defined as a dose administered within 24 hours of birth.

**
https://immunizationdata.who.int/listing.html?topic = coverage&location = eur

†† In Hungary, HepB is given at age 12 years, and in Slovenia, it is given at age 5–6 years.

§§ All countries are in northern Europe and have historically had very low HBV endemicity.

¶¶ Norway and the United Kingdom in 2017, and Switzerland in 2018.

*** Austria, Bosnia and Herzegovina, Germany, Montenegro, San Marino, and Ukraine.

††† Bosnia and Herzegovina and Russia do not report HepB-BD coverage.

§§§ The survey was sent to the 50 member states of EUR that implement universal HepB vaccination and included questions on HepB vaccination policy, practices, and measures to prevent perinatal transmission of HBV. Survey questions were designed to account for differences in HepB-BD policy between the member states (universal versus selective). Forty-three (86%) countries, including 22 of 23 countries with universal HepB-BD policy and 21 of 30 countries with selective HepB-BD policy, responded to the survey.

¶¶¶ Montenegro reported not having nationwide antenatal screening in place.

**** Six countries (Czechia, Estonia, France, Greece, Ireland, and Luxembourg) also had ≥90% coverage with routine HepB3 among infants each year during 2017–2019 (i.e., met the validation criteria for immunization).

†††† Croatia, Czechia, Denmark, Italy, Malta, the Netherlands, Slovakia, Switzerland, and the United Kingdom. Sources: the 2018 WHO Regional Office for Europe survey (Croatia, Czechia, Malta, Slovakia, and Switzerland); reports submitted to the WHO European Regional Hepatitis B Working Group (the Netherlands and the United Kingdom), https://www.sciencedirect.com/science/article/pii/S0264410X11016355?via%3Dihub (Denmark); https://www.sciencedirect.com/science/article/pii/S0163445310003518?via%3Dihub (Italy).

§§§§ Czechia, the Netherlands, Slovakia, Switzerland, and the United Kingdom; coverage was <90% only for Switzerland. Sources: the 2018 WHO Regional Office for Europe survey; reports submitted to the WHO European Regional Hepatitis B Working Group (the Netherlands and the United Kingdom).

¶¶¶¶ At the time of review of the validation documents by the WHO European Regional Hepatitis B Working Group, 3 years had not yet passed since introduction of universal HepB vaccination in the United Kingdom in mid-2017.
