# *Erratum*: Vol. 70, No. 30

**DOI:** 10.15585/mmwr.mm7033a6

**Published:** 2021-08-20

In the report “Progress Toward Hepatitis B Control — World Health Organization European Region, 2016–2019,” on page 1030, in the second column, first paragraph, the last sentence should have read, “Of the 21 countries with universal HepB-BD that reported birth dose coverage to WHO,^†††^ coverage with timely HepB-BD during 2016–2019 was ≥90% in **19–20** (90%–95%) **countries**.”

